# Incisionless facial resection for Kadish stage C olfactory neuroblastoma: Transcaruncular approach with combined endonasal and skull base surgery

**DOI:** 10.1002/ccr3.2906

**Published:** 2020-05-05

**Authors:** Tetsuya Ogawa, Kunihiro Nishimura, Yasuhiro Takahashi, Kenichiro Iwami, Tsuneo Yasumura, Kinga Yo, Hiroki Okamoto, Daisuke Inukai, Rui Sano, Tadashi Watanabe, Hirohiko Kakizaki

**Affiliations:** ^1^ Department of Otorhinolaryngology Aichi Medical University Aichi Japan; ^2^ Department of Oculoplastic Orbital & Lacrimal Surgery Aichi Medical University Aichi Japan; ^3^ Department of Neurosurgery Aichi Medical University Aichi Japan; ^4^ Department of Plastic surgery Aichi Medical University Aichi Japan

**Keywords:** endonasal, incisionless facial resection, Kadish stage C olfactory neuroblastoma, multidisciplinary team surgery, skull base surgery, transcaruncular approach

## Abstract

This case report describes resection without facial incision for aggressive Kadish stage C olfactory neuroblastoma (ONB). We performed resection via transcaruncular approach with combined endonasal and skull base surgery. This multidisciplinary team surgical approach is expected to lead to a new strategy for this type of tumor in the future.

## Graphical abstract

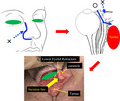

## 1 INTRODUCTION

Olfactory neuroblastoma (ONB) is a rare malignant tumor that derives from the neurosecretory cells of the olfactory epithelium.^1^ ONB represents approximately 3%‐6% of all malignant tumors of the nasal cavity.^2^ For anatomical reasons, ONB tends to invade directly into the nasal cavity, paranasal sinuses, and orbital area as well as intracranial tissue such as the dura and the brain.

**Figure 1:**
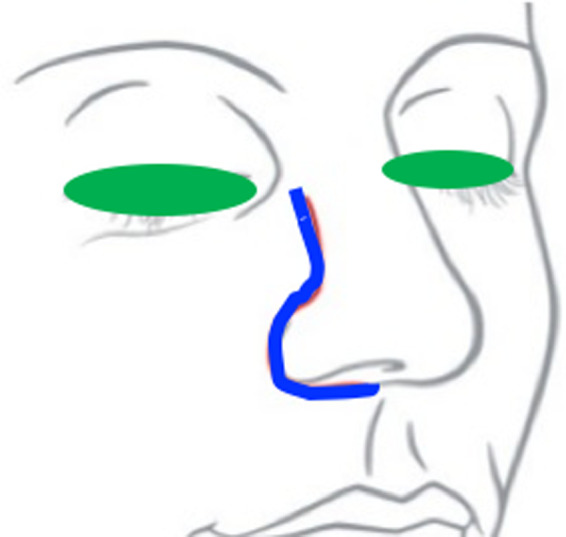
Lateral rhinotomy incision line

Surgery followed by radiotherapy is widely accepted for treatment.^3^ Recently, induction chemotherapy has been becoming an important modality for treating cases of aggressive and recurrent ONB.^4^, ^5^ However, definitive surgery has an important role in the treatment of ONB *En bloc* surgical resection is required to achieve complete curative resection of ONB, but this is sometimes difficult due to anatomic and aesthetic reasons. ONB quite easily invades the ethmoid sinus completely and sometimes nasal bone, and so facial skin incision is required to ensure complete resection, which is necessary to remove the medial orbital wall and nasal bone from the lateral and anterior external bone. *En bloc* resection is required in aggressive cases, usually via an anterior craniofacial approach combined with bifrontal craniotomy resection and a lateral rhinotomy or Weber‐Ferguson incision, as a standard surgical technique for complete resection.^3^ This strategy is good for aggressive surgical resection for ONB but it requires a facial skin incision (Figure 1). This sometimes impairs the patient's quality of life (QOL).

From this perspective, extended endoscopic endonasal surgery has recently been adapted for some cases of ONB for which this procedure is indicated.^6^, ^7^ This approach is adapted for some cases of aggressive ONB without need for facial skin incision, which ultimately results in shorter hospital stay and good QOL. Nonetheless, not all cases of ONB can be amenable to this procedure.

**Figure 2:**
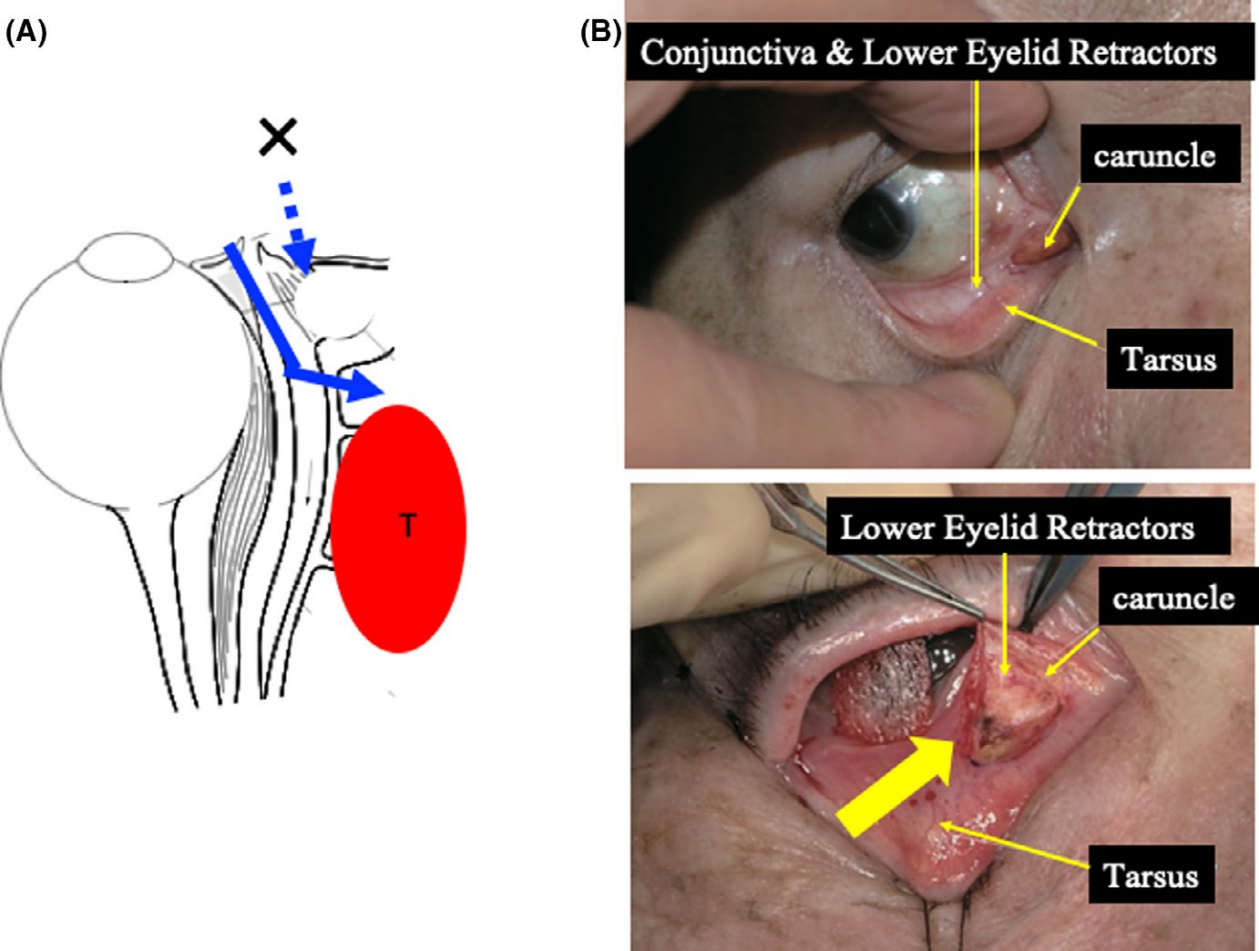
A: If the surgeon could approach directly in the orbit and attach the medial orbital wall from the lateral side, there would be a surgical margin at this point with no need for lateral rhinotomy or Weber‐Ferguson facial incision. Arrow: Line of approach without facial incision. T: Tumor. B: Transcaruncular approach for ocular surgery to allow adequate surgical field under direct vision

However, in the clinical setting, there are cases where the tumor has invaded the medial orbital wall. If the surgeon could approach directly in the orbit and attach the medial orbital wall from the lateral side, there would be a surgical margin at this point with no need for lateral rhinotomy or Weber‐Ferguson facial incision (Figure 2A).

Recently, the oculoplastic, orbital, and lacrimal surgery team at our institution reported their transcaruncular approach for eye surgery that affords a wide surgical field for direct visualization^8^ (Figure 2B). This is done by preserving the suspensory ligament of the eyeball, which passes into the posterior aspect of the eyeball. Using this technique, the medial orbital wall bordering the ethmoid can be seen completely under direct visualization even without a facial skin incision.

We encountered this case of ONB where the tumor had invaded the nasal cavity and ethmoid sinus with intracranial extension, but had not invaded the anterior aspect of the ethmoid sinus. Based on these findings, we opted to perform resection without a facial incision and using a transcaruncular approach with combined endonasal and skull base surgery. This method is expected to lead to a new technical concept of surgery ONB operation; thus, we here report this as a new technique for resection of ONB.

## 2 METHODS

### 2.1 Clinical findings and treatment strategy

**Figure 3:**
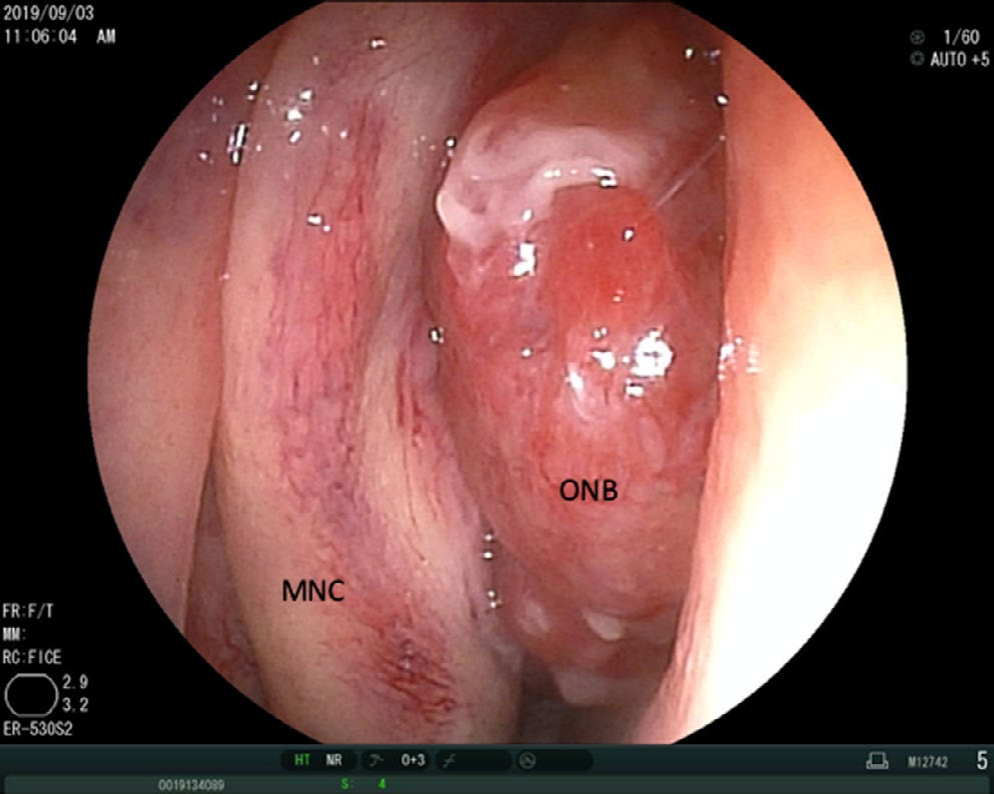
Endonasal flexible fiberoptic endoscopy reveals a massive tumor in the right nasal cavity. MNC, Middle nasal concha; ONB, Olfactory neuroblastoma

**Figure 4:**
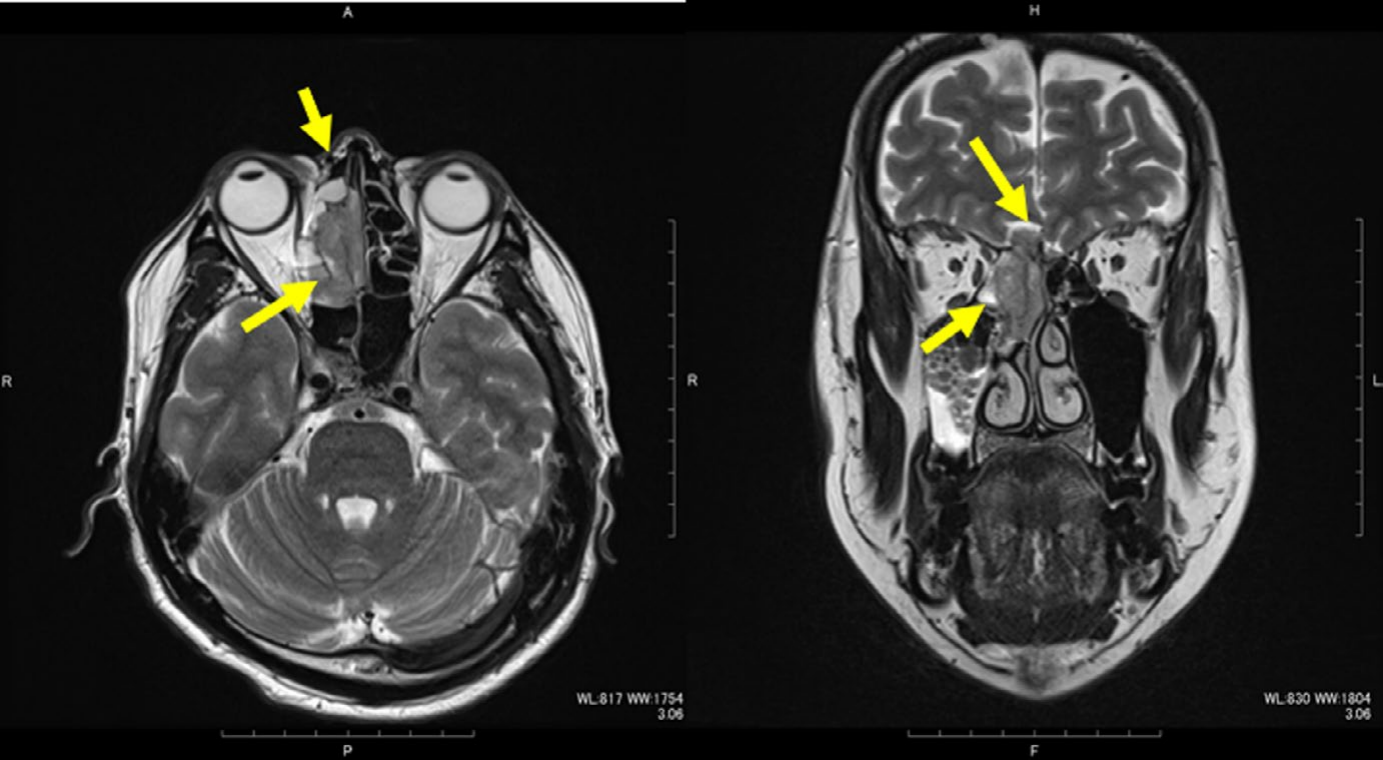
MRI findings. Left: Ethmoid sinus tumor seen attached to the right medial orbital wall but not invading the anterior aspect. Right: Tumor is attached to the right medial orbital and invaded to intracranial space

The patient was a 43‐year‐old man with a history of hyposmia who had been treated at another hospital. He was diagnosed with a right nasal tumor for which a biopsy was performed. He was then referred to our university hospital. On further investigation, endonasal flexible fiberoptic endoscopy revealed a massive tumor in the right nasal cavity (Figure 3). Pathological examination gave a final diagnosis of ONB, Hyams grading II. Computed tomography (CT) revealed the tumor with intracranial extension occupying the ethmoid and sphenoid sinuses. The tumor was seen to be attached to the right lateral wall of the ethmoid sinus, but had not invaded the anterior part of the sinus (Figure 4). Positron emission tomography‐computed tomography revealed no cervical lymph node or distant metastasis. The final diagnosis was ONB, Hyams grading II, Kadish C, T4N0M0.

Because the disease stage was considered to be progressive even though Hyams grading II, he was initially given neoadjuvant chemotherapy with etoposide and cisplatin. Evaluation after 1 cycle showed there was no change in size, and so our medical team decided to proceed with definitive surgery to remove the entire tumor.

### 2.2 Surgical plan

**Figure 5:**
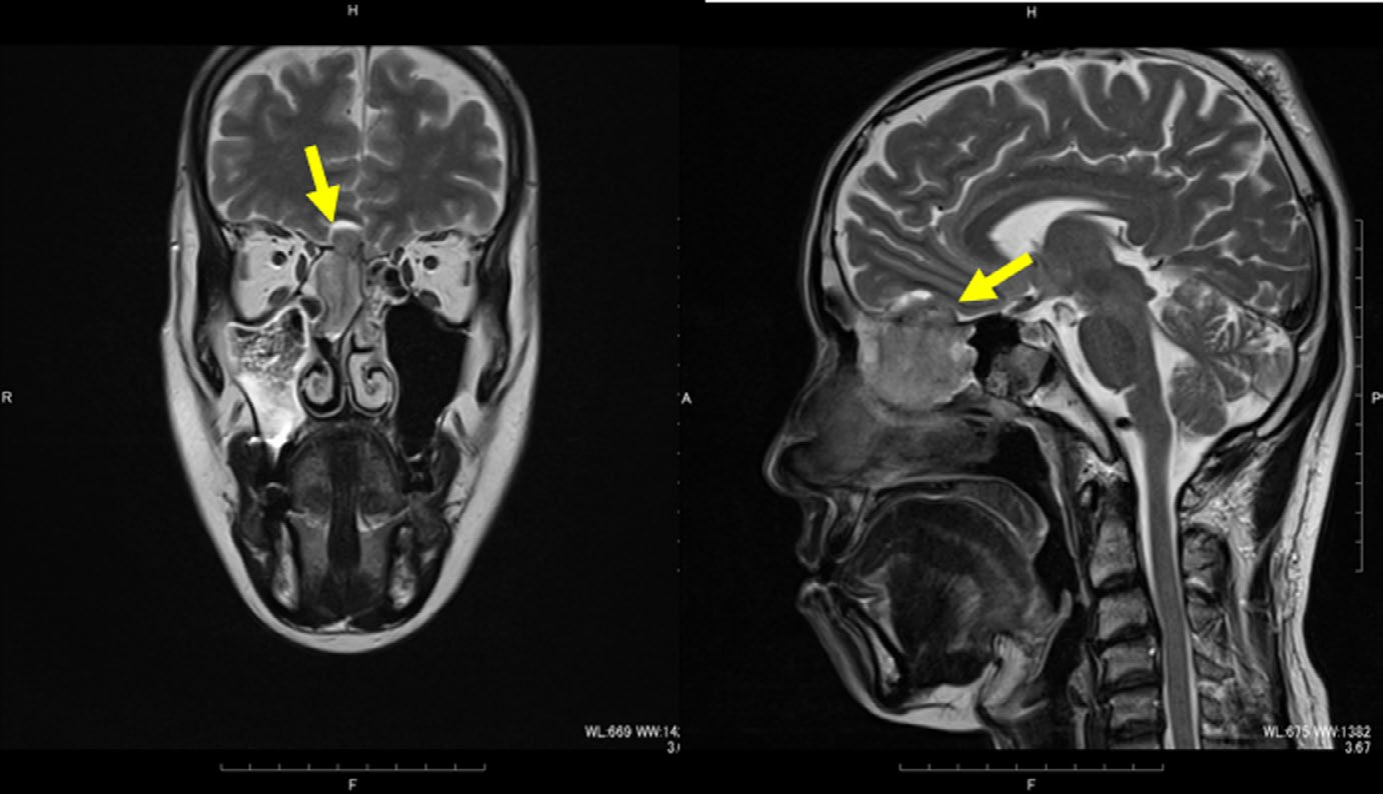
MRI image after neoadjuvant chemotherapy still showing intracranial invasion of the tumor to the brain and the most anterior aspect of the sphenoid sinus

After neoadjuvant chemotherapy, ONB status was intracranial invasion to the brain with anterior sphenoid sinus invasion (Figure 5). For complete resection, the oculoplastic, orbital, and lacrimal surgery, the endonasal surgery, and the skull base surgery team jointly performed multidisciplinary team surgery for definitive en bloc dissection. The surgical plan for each surgical team is described below.

1. Oculoplastic, orbital, and lacrimal surgery started with the transcaruncular approach to the face via the medial orbital wall of the ethmoid bone from the orbital cavity. This bone was considered the true lateral surgical margin of the tumor. This approach did not need a facial skin incision because the only incision was via the lacrimal caruncle on the periosteum just below the posterior lacrimal crest.
2. Endonasal surgery was performed by the otorhinolaryngologist. A sufficient and definitive surgical margin could be achieved using a nasal endoscope. A local nasal flap was raised to prepare a skull base covering. The entire laterally invading ONB tumor was resected by the oculoplastic, orbital, and lacrimal surgery team.
3. Skull base surgery was performed by the neurosurgeon from the superior aspect. The posterior aspect of the sphenoid sinus and the intracranial aspect of the tumor might be considered the true critical surgical margin for this tumor. This approach was successfully performed in joint surgery with the endonasal and the oculoplastic, orbital, and lacrimal surgeons extending from the skull base to the orbital cavity and the nose.
4. Plastic surgery was performed to reconstruct the skull base dural defect using fascia lata from the thigh. The otorhinolaryngologist then raised a periosteal pedicle flap, which was used to cover the nasal flap from the inferior aspect.

### 2.3 Surgical procedure

The patient was placed in the supine position. Airway intubation was initially performed via the oral cavity.

### 2.4 Transcaruncular approach

**Figure 6:**
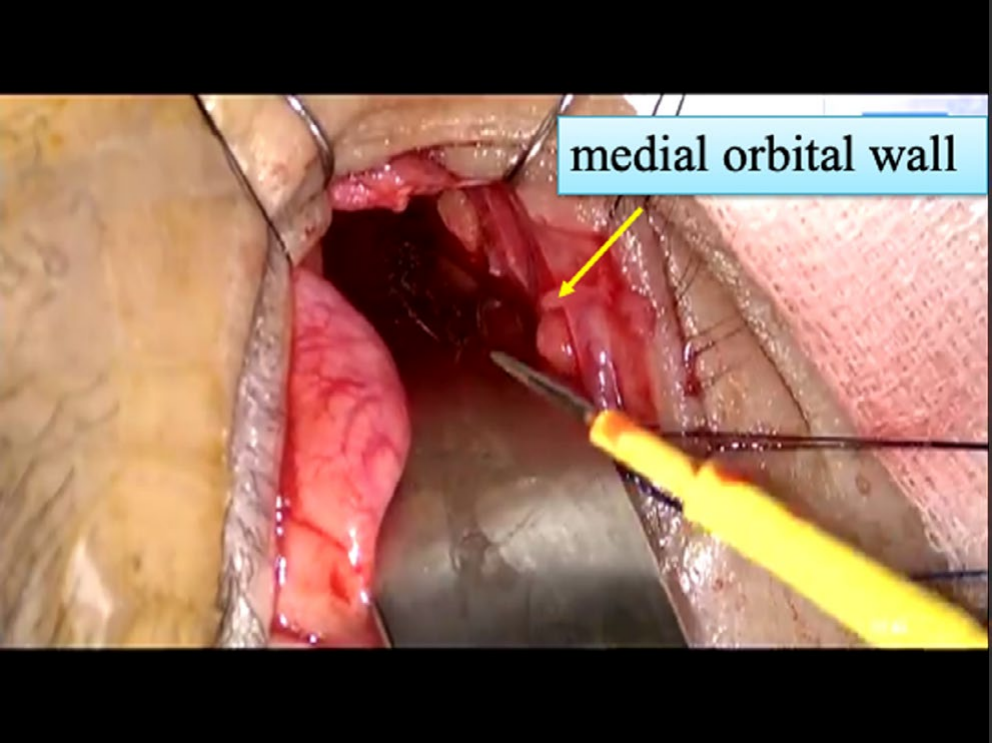
Incision made on the lacrimal caruncle using Westcott tenotomy scissors. Stevens tenotomy scissors were used for blunt dissection toward the medial orbital wall just below the posterior lacrimal crest; there was no incision of the facial skin

A right lacrimal incision was made by an oculoplastic, orbital, and lacrimal surgeon to approach the orbit. Another incision was made on the lacrimal caruncle using Westcott tenotomy scissors (Figure 6). Stevens tenotomy scissors were used for blunt dissection toward the medial orbital wall just below the posterior lacrimal crest. This incision was carried out on the periosteum just below the posterior lacrimal crest, but not on the facial skin. The periosteum was reflected from the medial orbital wall; then, the anterior and posterior ethmoidal arteries were cauterized. These steps allowed the gross tumor surface to be covered by this medial orbital wall. Also the periosteum around the medial orbital wall are totally preserved, the orbital compartment is not spread into this area. Then, the otorhinolaryngology team started the endonasal procedure.

### 2.5 Endonasal procedure

For the endonasal surgery, because the right lacrimal incision had already been made by the oculoplastic, orbital, and lacrimal surgeon, it was easy to transect the cauterized anterior ethmoid and posterior ethmoid arteries. It had already been confirmed that there was no tumor infiltration to the orbit, and so the median septum and its mucosa were elevated on the left side of the surgical field; there was no tumor invasion in this area. The sphenoid sinus had been converted into a single sinus and the interior was well‐aerated and there was no tumor invasion. The olfactory nerve thread was confirmed from both olfactory clefts; then, the frontal sinus was converted into a single sinus using an endoscopic modified Lothrop procedure (Draf III). The right frontal sinus was removed with a mucus retention cyst. The left side had good aeration. The right maxillary sinus was opened and was found to be intact. The medial surgical limit was determined by the left nasal septum mucosa, and the lateral orbital border was the vertical plate of the ethmoid bone. The lower aspect was a line bordering the anterior sphenoid sinus, inferior to the Vidian nerve externally, also for reducing the bleeding the Vidian nerve and sphenopalatine artery were transected. All rapid intraoperative margins were negative.

### 2.6 Skull base surgery

**Figure 7:**
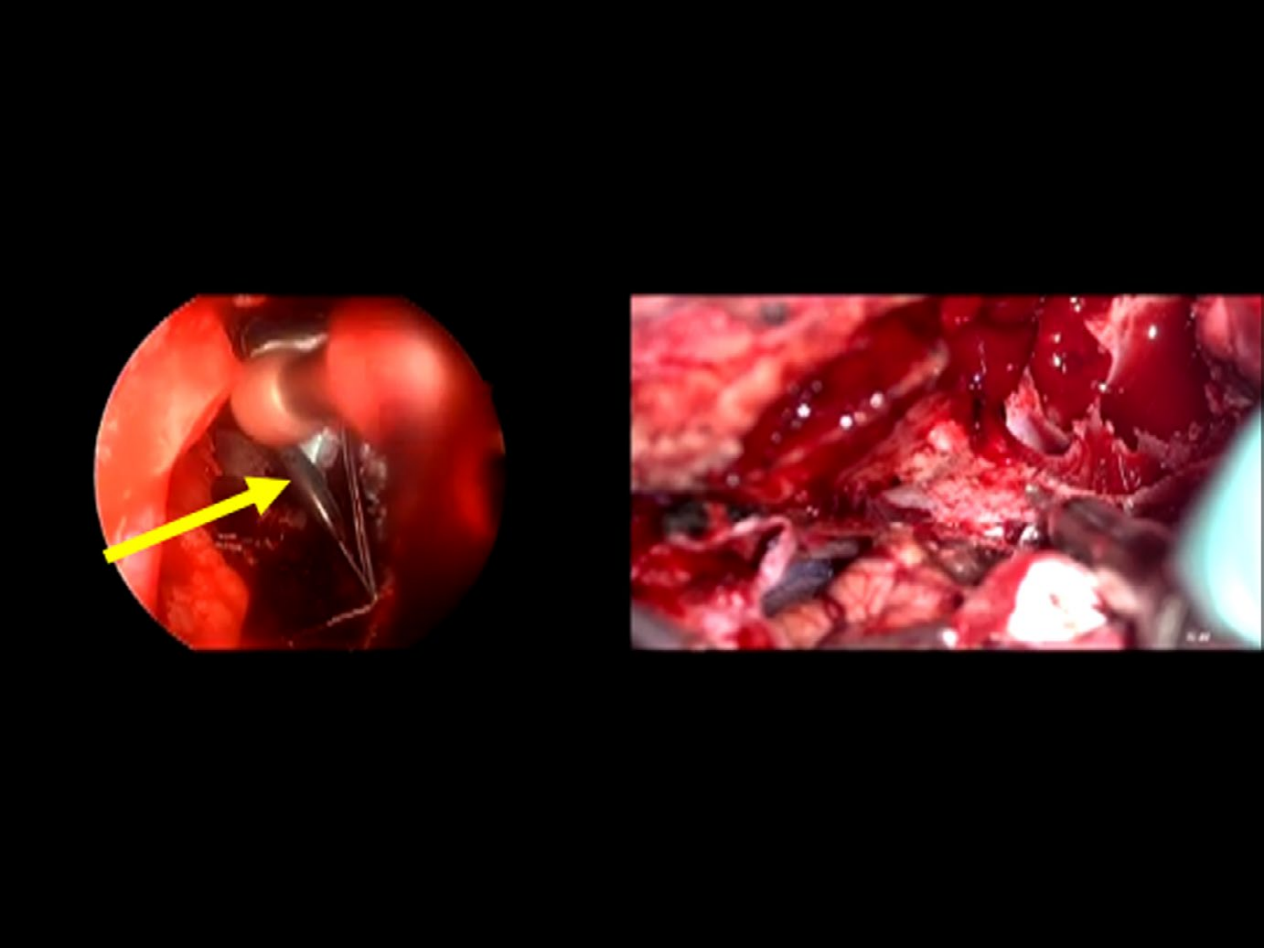
Right: the neurosurgeon could see the tumor distinctly with assistance from the endonasal surgeon. Left: the endonasal surgeon could approach the skull base via the sphenoid with assistance from the skull base surgeon. Arrow: Assistance from the neurosurgeon

Skull base surgery was performed by the neurosurgeon via a coronal incision placed slightly deeper than the hairline, following which craniotomy was performed. The anterior limit was confirmed in conjunction with a nasal endoscope, and this surgical margin was negative. They used a three‐dimensional video system and endoscope. The scalp was incised bilaterally via a coronal incision, and the flap was lifted off the periosteum up to the root of the nose. The facial nerve was preserved, and a periosteal pedicle flap was elevated for the skull base reconstruction. Bilateral frontotemporal craniotomy was performed with three burr holes. The dura was incised at the lower part of the forehead bilaterally and was cut with a cerebral sickle just above the cockscomb. An incision was made in the normal dura surrounding the tumor, exposing the anterior skull base bone. The right frontal sinus was cut and taken as tumor section. The tumor mass was mobilized in this order: osteotomy of right orbital wall → right optic nerve canal → sphenoid plane → left ethmoid sinus wall → left and right frontal sinus inferior wall. This was achieved by combining with endonasal surgery (Figure 7). The neurosurgeon could see the tumor from the nasal direction, and the endonasal surgeon could operate from the sphenoid with assistance from the skull base surgeon. Then ONB tumor was excised in its entirety from the head, nose, and eye. At this point, all surgical margins were negative.

### 2.7 Reconstruction for the skull base dural defect

The fascial flap was harvested from the right thigh by a plastic surgeon, the skull base was reconstructed with the periosteal flap and a thigh fascia flap of the frontal region, and a septal mucosal flap from the left side was used to cover the defect overlay.

## 3 RESULTS

### 3.1 Postoperative course

The postoperative course was uneventful, and oral intake was started on postoperative day 3 (POD3). There was no evidence of nasal, ocular, or intracranial infection. There were also no features suggesting cerebrospinal fluid leakage, and the patient was discharged from hospital on POD16. Examination revealed normal eye movement and position for both left and right eyes.

### 3.2 Pathology

Pathological examination revealed negative intranasal and ocular surgical margins. The most anterior part of the ethmoid sinus was also negative. However, intracranially most deep brain and sphenoid anterior tissue were close to the surgical margin.

### 3.3 Endoscopic findings

**Figure 8:**
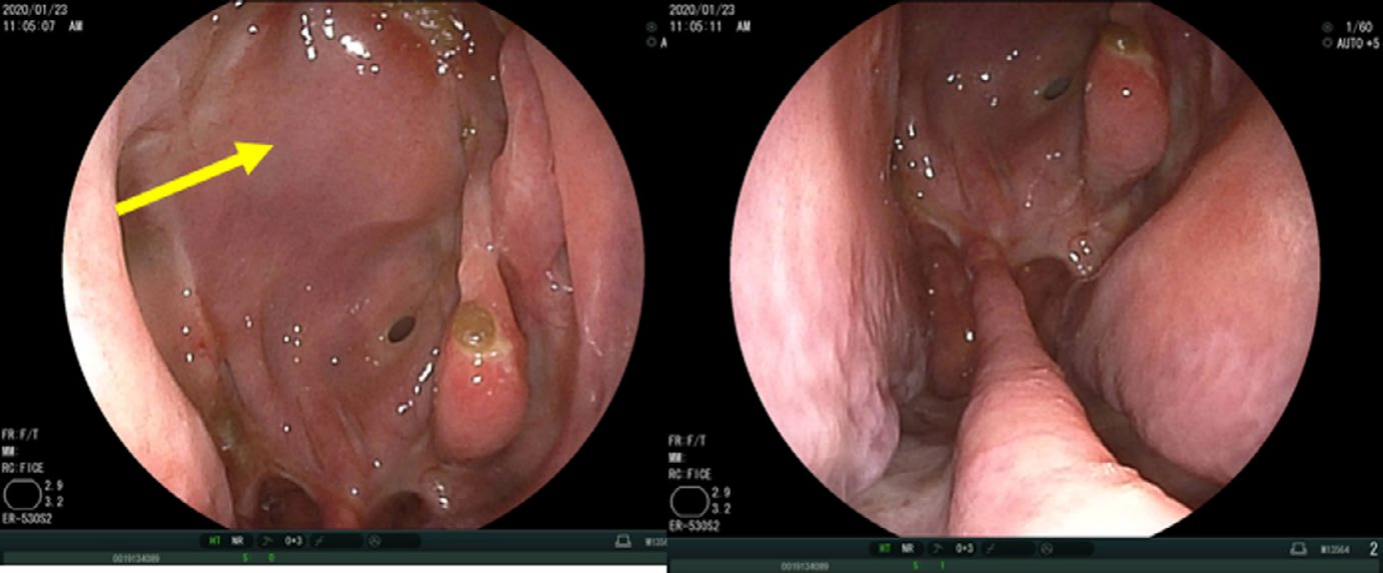
Postoperative nasal endoscopic findings after 4 mo. There is no cerebral spinal fluid leakage and no signs of infection. The reconstructed skull base mucosa covered by the nose mucosal flap is smooth, adapted, and well‐positioned on the nose. Arrow: Skull base

Postoperative nasal endoscopic findings after 4 months are shown in Figure 8. There was no cerebrospinal fluid leakage and no sign of infection. The reconstructed skull base mucosa covered by the nasal mucosal flap was smooth, adapted, and well‐positioned on the nose.

### 3.4 CT and magnetic resonance imaging findings

**Figure 9:**
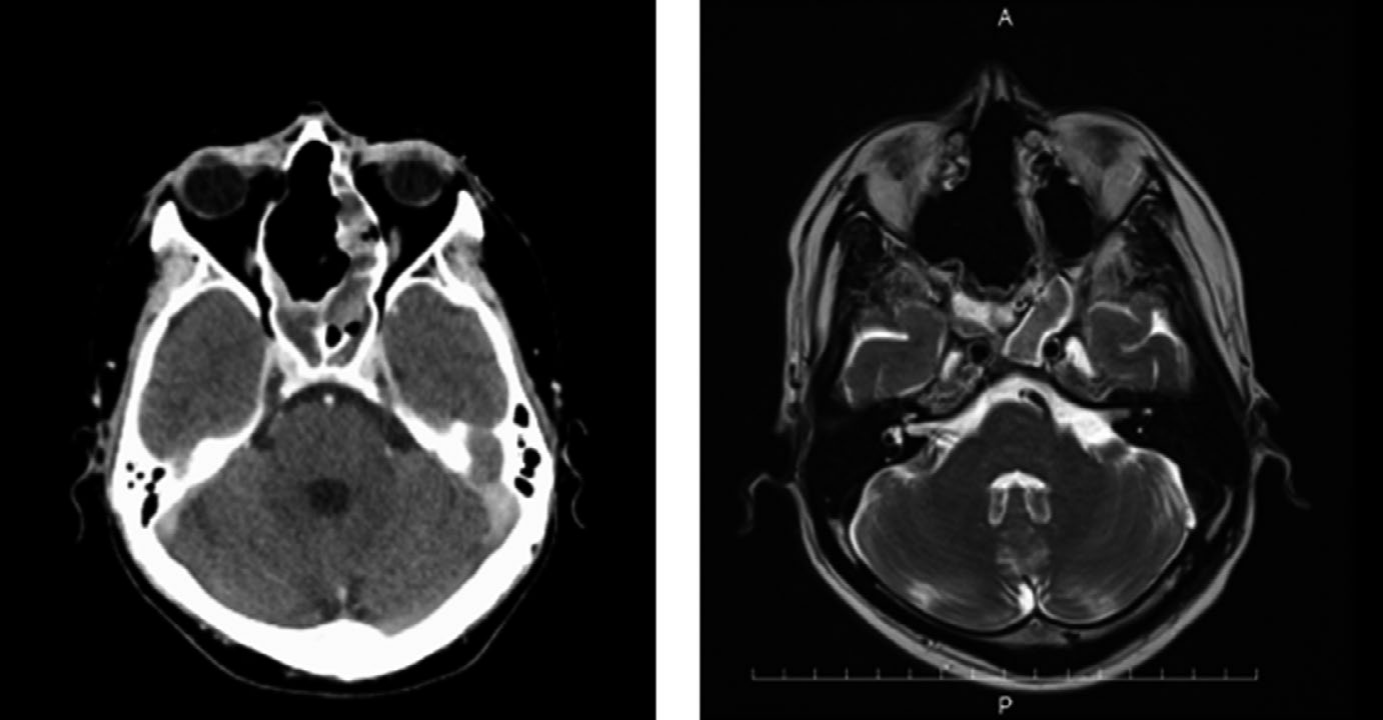
Computed tomography and magnetic resonance images showing complete tumor resection with preserved anterior ethmoid bone

**Figure 10:**
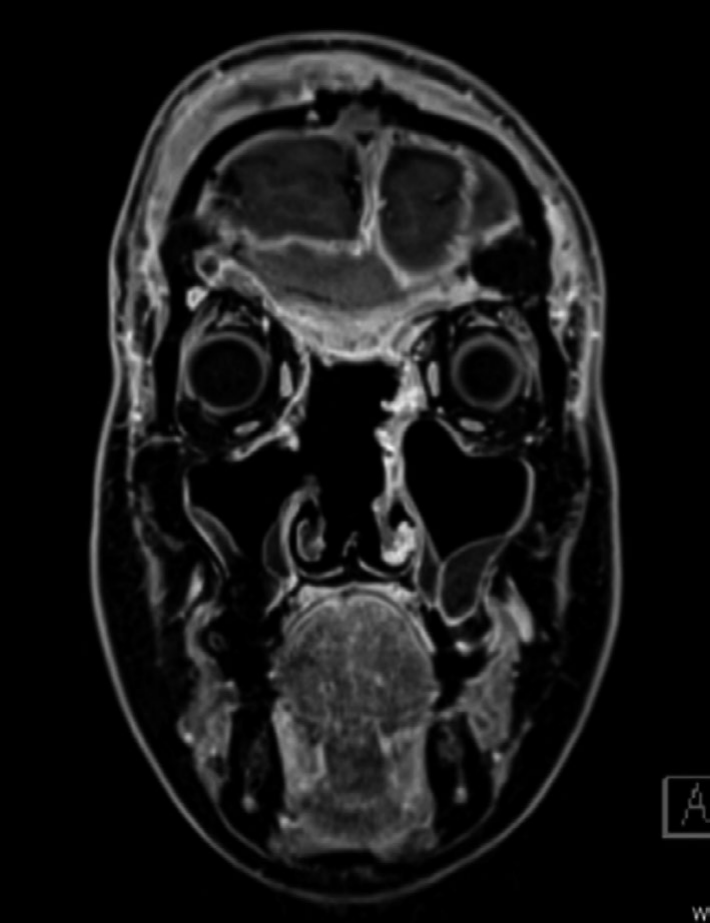
The extraocular muscles are also preserved and the position of the eyeball is stable. Skull base reconstruction was successful

CT and magnetic resonance imaging (MRI) showed that the tumor had been completely resected and the nasal bones and anterior ethmoid bone had been preserved (Figure 9). The extraocular muscles were also preserved, and the position of the eyeball was stable (Figure 10).

### 3.5 The face and eye movement

**Figure 11:**
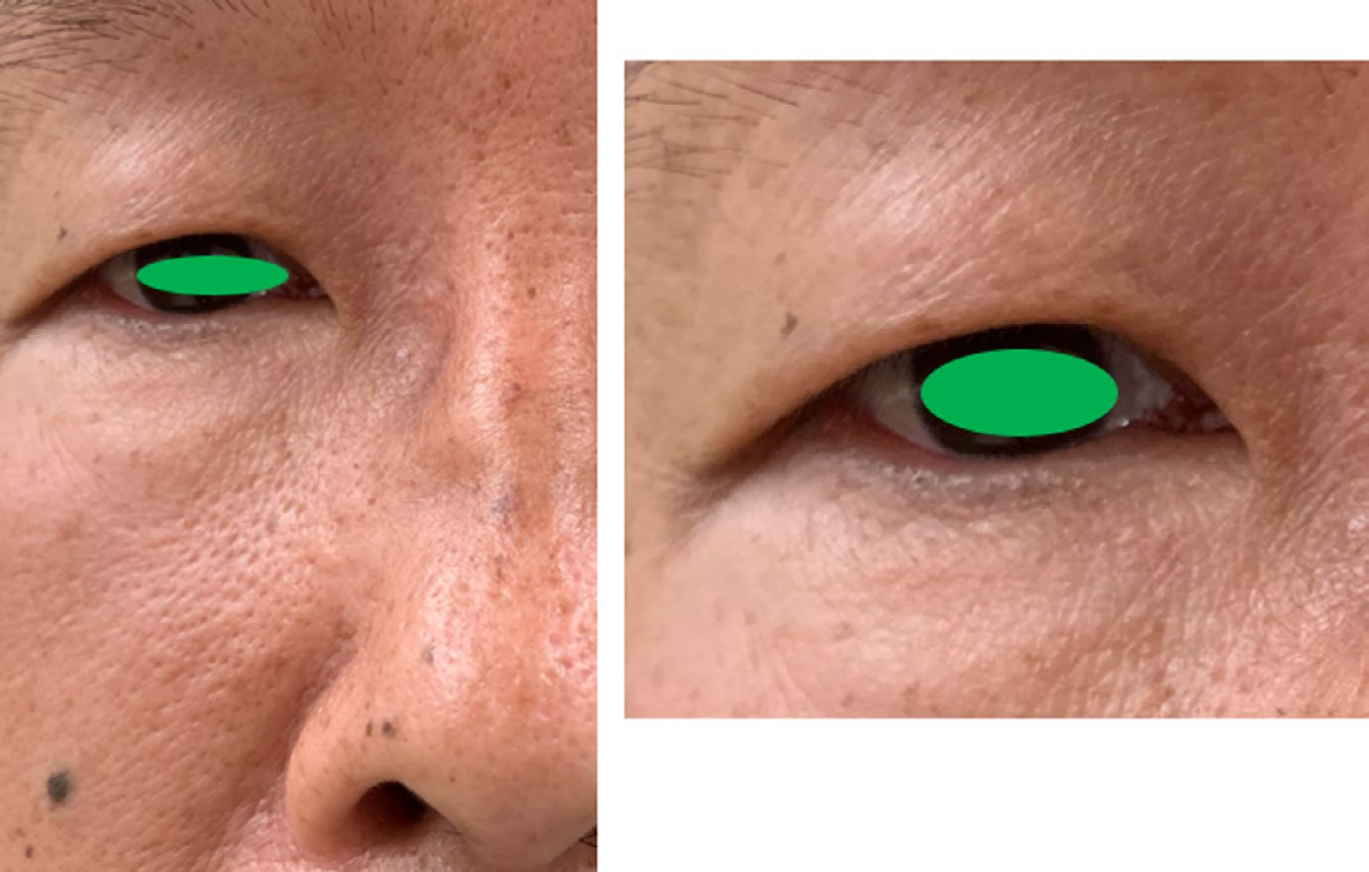
Image showing no skin incision on the face and no residual deformity of the nose and periorbital area eye. The incision line in the eye is not visible

There was no incision on the face and no deformity of the nose and periorbital area (Figure 11). The patient's orbital mobility is normal before surgery and also no changed after the surgery. Diplopia is not existed after the surgery.

### 3.6 Postoperative therapy

Because the brain and sphenoid sinus were close to the surgical margin, the tumor board meeting decided that the patient be given postoperative radiation therapy at a dose of 60 Gy. Preoperative PET‐CT shows no metastasis to the neck and also close margin areas are above only, radiation had done only local not included neck even though Kadish C, T4N0M0.^9^ Patient status has been checking every three weeks, and there was no sign of recurrence as of 7 months postoperatively.

## 4 DISCUSSION

Treatment of ONB is difficult, and surgery is the mainstay of treatment. If the tumor is small, minimally invasive surgery is possible, such as endoscopic surgery from only within the nose; however, such cases are rare. Because the tumor originates from the olfactory nerve, in many cases there is invasion to the brain and the sphenoid sinus. Recently, exclusive endoscopic transnasal approach is adapted for the treatment of sinonasal tumors including ONB even in case of advanced T4 stages.^10^, ^11^ Despite this endoscopic approach has been progressing, still now an intracranial surgical approach is sometimes required to address these aggressive cases, which is typically skull base surgery that requires joint surgery between an otorhinolaryngologist and a neurosurgeon. Also, for anatomical reasons, the tumor can easily invade the nasal bone and ethmoid sinus. Resecting this invading tumor requires direct access to the nasal and ethmoid bones via an anterior approach because these bones represent the true surgical margin. This procedure needs a facial skin incision.

Thus, en bloc resection for these aggressive cases requires an anterior craniofacial approach with combined bifrontal craniotomy resection and lateral rhinotomy or Weber‐Ferguson incision, and this is considered standard surgical technique for complete resection.^3^

Also, from the perspective of medical oncology, other aspects of anticancer drug treatment are warranted that can be adapted for ONB. This leads to better prognosis facilitation of minimally invasive surgery once tumor volume is decreased. This ultimately leads to improved QOL. Recently, the treatment strategy for ONB has been changed to take on a comprehensive approach.

In our case, the patient received neoadjuvant chemotherapy but with no improvement. We decided that radical surgery was necessary because the ONB status was T4, Kadish C. We initially planned surgery via an anterior craniofacial approach with combined bifrontal craniotomy and lateral rhinotomy or Weber‐Ferguson incision because of the aggressive nature of the tumor, which had invaded the brain and sphenoid sinus. On gross examination of the ethmoid sinus, the tumor had invaded but not extended to the anterior aspect, and a cavity was seen in the anterior ethmoid (Figure 4). Therefore, we considered that if we could fill this cavity with bone, we could then achieve a sufficient surgical margin with no facial incision (Figure 2). Our medical team has the skill and experience to access this area without skin incision via the transcaruncular approach; we thus used this method that required only a lacrimal incision and approached into the orbit without incising the skin. We believe this approach was suitable for this stage of ONB status with ethmoidal involvement.

Although joint surgery between eye and nasal surgeons is not popular, we have immense experience in collaborative surgery between the oculoplastic, orbital, and lacrimal surgery team and our endonasal surgery team,^12^ and we successfully resected the tumor in this area using a navigation system. This multidisciplinary team approach shows the benefits of joint surgery and the possibility of combining oculoplastic, orbital, and lacrimal surgery with endonasal surgery. We thus presumed this patient would benefit from multidisciplinary team surgery.

Furthermore, collaboration between the endonasal surgeon and the skull base neurosurgeon is also important and more common. We have previously reported successful teamwork between endonasal and skull base neurosurgeons at our institution.^13^ So, this novel multidisciplinary operation was a complete success with a combination of three highly skilled and experienced teams. To our knowledge, this is the first report of a multidisciplinary surgery comprising three teams. The strategy of facial incisionless resection for Kadish C ONB via a transcaruncular approach with combined endonasal and skull base surgery was useful and improved the patient's QOL.

Further on QOL, this technique seems to be beneficial for the patient because it avoids facial incision, which is good for maintaining QOL. Ng et al reported that a skin incision on the face is a major concern of most patients and concluded that facial incisionless surgery is preferable for patients.^14^ In this patient, the ONB had invaded the brain and sphenoid sinus extensively, we could perform a procedure that maintained the patient's QOL like asfacial incisionless resection via a transcaruncular approach with combined endonasal and skull base surgery. Surgical oncologists should consider a treatment strategy that maintains QOL even for very aggressive cases such as T4 and Kadish stage C. As surgical oncologists, we must consider QOL in terms of treatment options especially for aggressive cases.

In general, for treatment of highly aggressive ONB, surgeons might easily opt to use an anterior craniofacial approach with combined bifrontal craniotomy resection and lateral rhinotomy or Weber‐Ferguson incision. If the tumor had invaded the nasal cavity and ethmoid sinus but had not invaded the anterior aspect of the ethmoid sinus, we believe that transcaruncular approach has an advantage for getting the exact surgical margin from the outside of the tumor. Even though very aggressive cases such as T4 and Kadish stage C, three‐team surgery would allow for possible complete resection without facial incision. Therefore, our novel approach to facial incisionless resection for this type of ONB via a transcaruncular approach with combined endonasal and skull base surgery is a new technique for resection of this type of tumor. Further investigation and improved treatment are expected in the future.

## CONFLICT OF INTEREST

None declared.

## AUTHOR CONTRIBUTIONS

TO, HK, and TW: involved in surgical concept. KN and KY were the main surgeons for nasal surgery. HO, DI, and RS were ENT surgery assistants. YT: involved in eye surgery. KI: involved in brain surgery. TY: involved in reconstructive surgery.
